# Enhancing Detection of Feline Chronic Kidney Disease Through Smart Litter Box Monitoring

**DOI:** 10.3390/ani16091319

**Published:** 2026-04-25

**Authors:** Natalie Langenfeld-McCoy, LeAnn Snow, Heidi Gordon, Zachary George, Jessica Quimby, Olivia Arndt, Sarah Thomas, Nicholas Schoeneck, Ragen T. S. McGowan

**Affiliations:** 1 Nestlé Purina Research, Checkerboard Square, St. Louis, MO 63164, USA; leann.snow@rd.nestle.com (L.S.); heidi.gordon@rd.nestle.com (H.G.); olivia.arndt@rd.nestle.com (O.A.); sarah.thomas@rd.nestle.com (S.T.); nicholas.schoeneck@rd.nestle.com (N.S.); ragen.trudelle-schwarzmcgowan@rd.nestle.com (R.T.S.M.); 2 Veterinary Clinical Sciences, College of Veterinary Medicine, The Ohio State University, Columbus, OH 43210, USA; zgeorgevet@gmail.com (Z.G.); quimby.19@osu.edu (J.Q.)

**Keywords:** chronic kidney disease (CKD), feline elimination behavior, litter box monitor, supervised machine learning, smart pet lot devices, health monitoring, CKD detection

## Abstract

Chronic kidney disease (CKD) is a prevalent condition in cats and recognized as a leading cause of mortality among cats generally, yet it is difficult to detect by cat caregivers, especially in its early stages. This retrospective study aimed to enhance detection of CKD by cat caregivers through a smart litter box monitor technology that tracks feline elimination behaviors. We established cohorts of cats with CKD and cats with no known conditions and analyzed behavioral features captured by the monitor. A mixed-effects model identified significant differences, which were then integrated into a machine learning framework for predictive modeling. The model achieved a weighted F1-score of 92.7% in training using cross-validation and 89.9% in validation, demonstrating high precision for CKD predictions. Key findings included a behavioral profile unique to cats with CKD characterized by increased urination frequency, longer elimination durations, and reduced post-elimination covering behavior. These results suggest that the smart litter box monitor can provide valuable, continuous, and non-invasive data for CKD detection and management.

## Simple Summary

Chronic kidney disease (CKD) is a common and serious health issue in older cats that is irreversible and often challenging to detect, especially in the early stages of the disease. This study investigated how a new technology, the smart litter box monitor, could help detect behavioral changes related to CKD by tracking how cats use the litter box. We compared cats with CKD (Renal group) to a group of similar age and sex matched cats with no known conditions (Non-Renal group), analyzing their elimination patterns. The findings showed that the Renal group urinated more often, spent a longer time eliminating, and covered their waste with less force and for a shorter duration than the Non-Renal group. The smart litter box monitor was able to detect these behaviors and identify them as unique to this group of cats. This could help caregivers and veterinarians identify CKD and manage the condition better because the monitor offers an in-home, ongoing 24/7 passive data collection of elimination behavior trends and changes.

## 1. Introduction

Chronic kidney disease (CKD) is one of the most common irreversible, and often progressive, syndromes experienced by pet cats [1,2,3,4]. Estimates range from 31% in cats > 15 years old [1] to 30–40% of cats > 10 years old [5] and it is considered a primary cause of mortality in cats overall [6,7,8]. There is also evidence of higher prevalence: one study found CKD in 50% of randomly selected cats aged 6 months to 20 years [9]. Despite the high prevalence, signs of CKD are often missed by pet caregivers because they may not know what changes to look for, or because even known behaviors are difficult to reliably observe and track. Moreover, clinical diagnosis presents a substantial challenge for veterinarians who have limited visibility into a cat’s behavior at home and typically only encounter patients at annual visits or once overt signs have advanced sufficiently to raise caregiver concern.

Some of the literature suggests that CKD, in its early stages, is characterized by clinical changes that are present but typically too subtle for caregivers to readily detect [10]. As such, there is general interest in studying risk factors, biomarkers, and detection mechanisms to better understand and treat CKD as early as possible [1,2,5,6,11,12]. Due to the complexity of the syndrome, timely detection and diagnosis of CKD can be difficult [11,13]. Diagnosis of CKD is further complicated by the presence of non-specific clinical signs (i.e., weight loss, dehydration, vomiting, altered appetite) and comorbidities (i.e., hyperthyroidism, lower urinary tract disease) [6,10]. In one cohort case study [14], the most frequent clinical signs preceding diagnosis were reported to be weight loss (49.9%; 311), excessive thirst (polydipsia) (38.6%; 241) and excessive urination (polyuria) (24.5%; 153). At diagnosis, over 56.6% of the cohort cats (354) had two or more compatible clinical signs recorded, while nearly 25% (155) had no clinical signs recorded [14].

Biomarkers hold promise as possible sources of early detection; however, they also hold inherent limitations like lacking a single salient marker for assessment, non-renal factors, poor sensitivity required for reliable early detection, misinterpretations due to wide reference ranges, and lacking robust validation [5,6,7,8,11,15]. Overall, routine blood and urine testing, in combination with medical histories and clinical findings, are considered the most reliable means for screening kidney disease [3,8,10,16]. And still, up to 75% of functional renal mass may be lost before azotemia (persistently increased creatinine and BUN concentration) is detected by testing [17].

Algorithms and machine learning have started to be leveraged with promising results as a possible tool for the detection of CKD, in some cases combining biomarkers and risk factor features in a recurrent neural network (RNN) [15], and in others evaluating predictive confidences of multivariate biomarkers using random forest (RF) and support vector machine (SVM) modeling to detect up to 6 months earlier than typical diagnosis [8]. Digital tools may contribute enhanced detection and may also facilitate managing the chronic, progressive course of CKD in which regular monitoring is widely acknowledged as essential for optimizing the efficacy of interventions [3]. This is one of the emerging benefits of the smart litter box monitor, a device that passively collects continuous longitudinal data of a cat’s elimination patterns [18]. The load cell sensors imbedded in the smart litter box monitor detect signals related to cat behaviors in and around the litter box. AI models were rigorously trained by a supervised machine learning methodology in which hundreds of thousands of events time-synced with video footage were labeled. The system predicts cat or human interaction, the type of cat event that is occurring (i.e., urination, defecation, non-elimination), specific cat IDs within a multi-cat household, and weight measurements, among other features related to cat elimination behaviors [18]. The smart litter box monitor also alerts cat caregivers to changes in cats’ weight and elimination behaviors. Due to the signal detection capabilities of the smart litter box monitor and its alerting capabilities, this device may support caregivers by providing health- and behavior-related insights.

For example, a group of researchers have identified elimination differences (both urination and defecation) among cats with CKD [1] using the same smart litter box monitor technology [18]. This 2025 study found that cats with CKD had a higher mean number of urination events per day compared to the healthy cat group. This same study was primarily focused on defecation patterns and found that CKD cats defecate less frequently than healthy cats. More specifically, cats with CKD had a lower mean number of defecation events per day over the 30-day study period, and CKD cats defecated less frequently per day and aggregated across the observation periods. This study provided the first published evidence for the capabilities of the smart litter box monitor to assess meaningful changes as related to CKD.

The present retrospective study aims to demonstrate that the smart litter box monitor may be a useful tool empowering cat caregivers and veterinarians with AI-driven data collected passively, continuously, and noninvasively for CKD detection. The aim of this study was to synthesize complex elimination behavior features from a smart litter box monitor and develop a predictive model for the detection of CKD. We hypothesized that we could detect behavioral differences between cats with CKD (Renal group) and cats with no known conditions (Non-Renal group) using features from sensors within the smart litter box monitor technology, and that these features would support the development of a predictive model to identify cats exhibiting a unique behavioral profile indicative of CKD.

## 2. Materials and Methods

This retrospective study analyzed data collected from January 2023 to September 2025. The dataset utilized in this study was compiled from two primary sources: the Nestlé Purina PetCare Center (NPPC) in Missouri, USA, and various in-home (IH) environments. Cats were categorized into two groups, termed Renal and Non-Renal. The NPPC cats in the Renal group were all diagnosed with CKD by a veterinarian and receiving veterinary standard of care for CKD. Diagnosis of CKD was based on creatinine greater than 1.6, blood urea nitrogen greater than reference range, and USG < 1.035, in combination with patient history and physical exam findings. The sample of IH cats comprised two groups: (1) clinical study cats that had confirmed veterinary diagnoses of CKD and receiving CKD standard of care [1]; and (2) general population cats, categorized based on caregiver-reported CKD diagnoses with unknown treatment status.

The data from this retrospective study had been collected under Institutional Animal Care and Use (IACUC) approval or informed consent of data usage. The Nestlé Purina IACUC and Ohio State University IACUC numbers were NT9913 and IACUC-2023A00000032, respectively. For all IH data collection, signed informed consent waivers were gathered. Additionally, caregiver-reported health information was obtained from a voluntary in-app health survey from cat caregivers utilizing the smart litter box monitors and contributed a group of cats with reported “no known health conditions” which were included in the Non-Renal group.

Cats from this data pool were split into two groups for modeling purposes: a Leave-One-Out Cross-Validation (LOOCV) set was used for training the model, and a second group, called the validation set, was used to evaluate how the model would perform on unseen cats. The training cats included the 13 CKD cats (4 from NPPC and 9 from IH) with confirmed International Renal Interest Society (IRIS) stage (stage 1 (*n* = 2), stage 2 (*n* = 4), stage 3 (*n* = 5), stage 4 (*n* = 2)) and 72 cats (22 from NPPC and 50 from IH) with no known conditions. For each cat in the Renal group there was a cat of similar age in the Non-Renal group, but the Non-Renal group had a wider range of ages and a lower average age (see Table S1). The validation Renal group comprised 7 cats (1 from NPPC and 6 from IH) with reported CKD and 44 cats (3 from NPPC and 41 from IH) with no known conditions. IRIS stage was not recorded for all cats in the Renal group validation set, thereby simulating a real-world use case of the smart litter box monitor technology. However, the inability to confirm self-reported diagnoses of the IH cats in the validation set presents a limitation and added uncertainty. Each cat in both the training and the validation group contributed multiple weeks of data from the smart litter box monitor technology. Data ranged from a minimum of 2 weeks to a maximum of 104 weeks (median: 6 weeks) for each cat. To prevent data leakage from the multiple weeks of data, LOOCV was employed at the cat level, grouping all weekly observations from each cat so that individuals appeared exclusively in either the training or test set within a fold. The dataset was partitioned into 13 folds, each containing one renal cat and a subset of non-renal cats, ensuring that no observations from the same cat were shared between training and testing sets.

**Figure 1:**
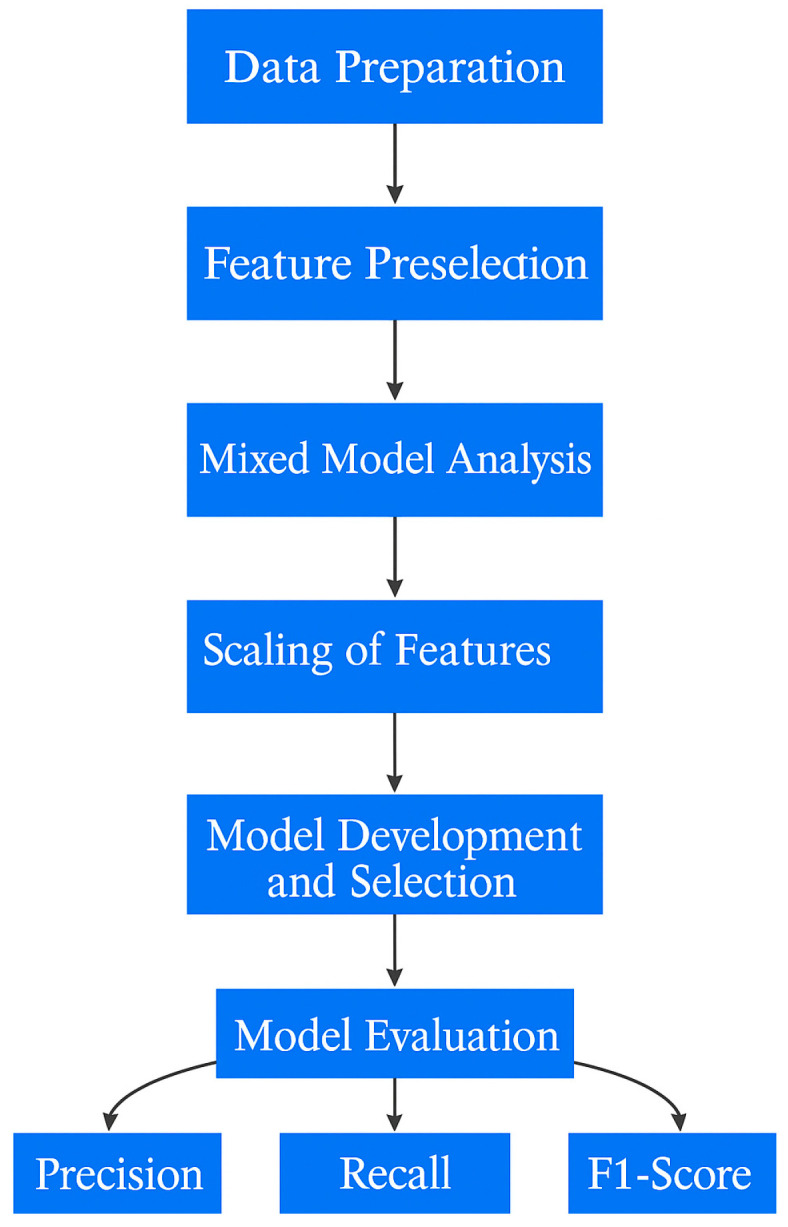
Model development process.

To begin the data preparation, data filtering criteria were established to include weeks with a minimum of four days of recorded data to ensure inclusion of households with cats actively using the smart litter box monitor. Outlier measurements of the urination and/or defecation weight of output were identified and removed, specifically those below −150 g or above 350 g. Combo events, defined as urination and defecation occurrences within a single elimination event, were aggregated into respective counts. Exploratory Data Analysis (EDA) was conducted by aggregating features at the weekly level for each cat, with modeling features computed using a 7-day rolling window. The EDA identified a wide initial pool of 120 potential statistical features (e.g., mean, medians, totals) from the available smart litter box monitor data so a feature preselection process was implemented to eliminate redundancy and retain only distinct features to use for the mixed model analysis step in the process, as shown in Figure 1.

Ultimately, 24 explainable features that could be measured by the smart litter box monitor were selected for further analysis to both validate known behavioral differences and uncover new differences between the two groups, including the following:

- Mean Daily Visits in a Week;
- Mean Transition/Covering Duration in a Week;
- Mean Transition/Covering Duration of Urination Events in a Week;
- Mean Elimination Duration of Defecation Events in a Week;
- Mean Event Duration in a Week;
- Sum of Weight of Output of Urination Events in a Week;
- Mean Elimination Duration of Urination Events in a Week;
- Mean Event Duration of Defecation Events in a Week;
- Mean Elimination Duration in a Week;
- Mean Covering Up Intensity of Urination Events in a Week;
- Mean Event Duration of Urination Events in a Week;
- Mean Entry and Digging Duration in a Week;
- Mean of Weight of Output of Defecation Events in a Week;
- Mean Transition/Covering Duration of Defecation Events in a Week;
- Mean Event Duration of Non-Elimination Events in a Week;
- Sum of Weight of Output of Defecation Events in a Week;
- Mean of Weight of Output of Urination Events in a Week;
- Mean Entry and Digging Duration of Urination Events in a Week;
- Standard Deviation of Weight in a Week;
- Mean Digging Up Intensity of Urination Events in a Week;
- Mean Daily Non-Elimination Events in a Week;
- Mean Entry and Digging Duration of Defecation Events in a Week;
- Mean Daily Defecation Events in a Week.

A mixed-effects model was then employed to identify features significantly associated with the two groups (Renal vs. Non-Renal), accounting for individual variability across cats. Mixed-effects models were conducted using R Software (v4.4.1) [19] and glmmTMB (v 1.1.12) packages [20]. The model was first structured as Model v1: Feature ∼ Condition + (1|pet_id).

However, the litter box maintenance practices at NPPC involved daily scooping, whereas in-home maintenance revealed that 25% of boxes were scooped daily, another 25% once a week, and 50% between 2 and 6 times per week. Previous research indicates that litter box maintenance can significantly influence behavior [21,22], necessitating the inclusion of Source as a control variable in the mixed models. To identify features impacted by Source, a second model was structured as Model v2: Feature Source + (1|pet_id).

Finally, to ascertain which features exhibited a statistically significant relationship with the health condition while controlling for environmental influence (Feature ∼ Source), the mixed-effects model that included Condition, Source, and the interaction fixed effects was deployed Model v3: Feature ∼ Condition + Source + Condition:Source + (1|pet_id).

To mitigate environmental impacts on key features that were significantly related to Condition, standardization was performed on those features that were also significantly related to the environment, scaling feature values within the NPPC and IH groups independently.

Significant features identified through mixed modeling analyses were inputted into a predictive modeling framework for further refinement. Modeling was conducted in Python (v.3.12.9) [23] using common machine learning packages (numpy 2.1.3; pandas 2.3.0; catboost 1.2.10; scikit-learn 1.5.1) [24,25]. A CatBoost model was constructed, chosen for its effectiveness in handling categorical variables and robustness against overfitting [26] and performed better (28% lift in Renal precision) than more simple models like logistic regression. Given the limited number of cats with CKD in the dataset, LOOCV was utilized for model training and testing. This approach maximizes the use of limited data, providing a more accurate estimate of model performance while reducing bias and detecting overfitting.

**Figure 2:**
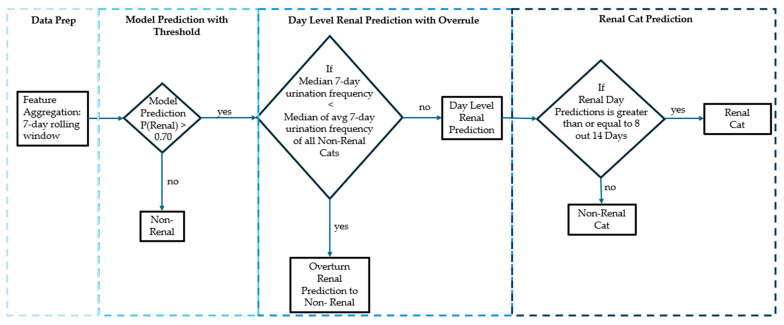
Flow for predicting Renal.

In predictive modeling, a common threshold for positive predictions is 0.5; however, a threshold of 0.7 was employed in this analysis to enhance precision (Figure 2). We systematically evaluated thresholds of 0.5, 0.6, 0.7, and 0.8 and found that a threshold of 0.7 provided the most favorable balance between precision and F1-score for the renal class. This elevated threshold reduces the likelihood of false positives, particularly critical in medical contexts.

Additionally, a Renal prediction override mechanism was established, requiring an elevated number of urination events over a 7-day rolling window to further refine predictions. The trained model generates daily predictions based on data collected over the preceding 7 days. A cat is classified as having CKD if it is predicted in the Renal group for 8 out of 14 consecutive days, allowing for variability in the data and reducing the risk of misclassification due to transient conditions.

To optimize model performance, Recursive Feature Elimination was employed, systematically removing the least significant features and re-evaluating the model’s performance iteratively to select for the most salient features (Table 1).

The performance metrics assessed included precision (true positive predictions), recall (sensitivity), and F1-score, with a focus on optimizing precision for the Renal group and recall for the Non-Renal group. Misclassification analyses were performed for incorrect predictions.

## 3. Results

Several significant elimination behavior differences among cats with CKD were observed, particularly regarding their urination events and related in-box activities (Table 2).

**Figure 3:**
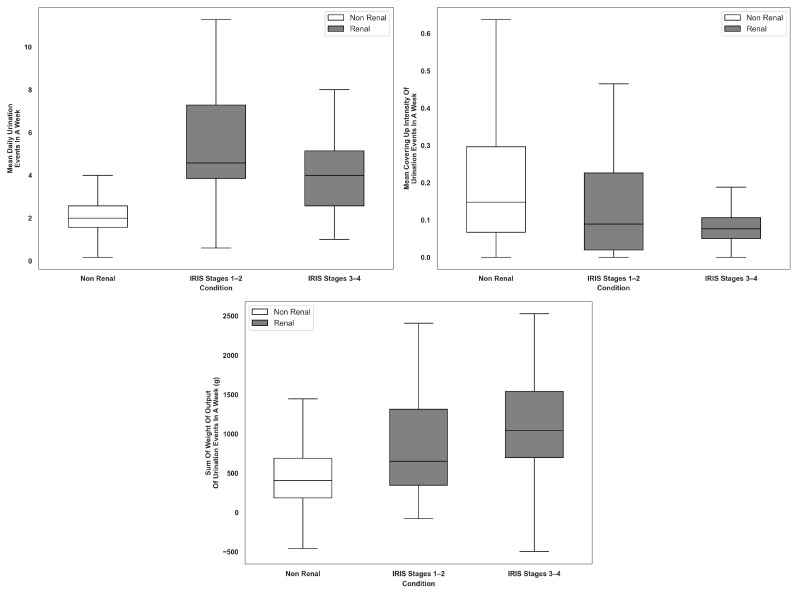
Boxplots of non-duration-based features for Non-Renal and Renal by IRIS stages for significant features.

**Figure 4:**
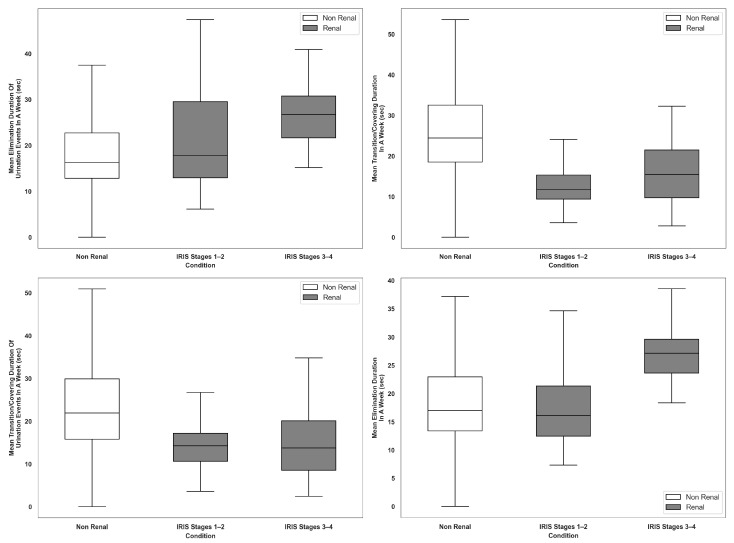
Boxplots of duration-based features for Non-Renal and Renal by IRIS stages for significant features.

Cats in the Renal group demonstrated an increased frequency of daily urination events compared to cats in the Non-Renal group with a mean of 4.6 events per week for cats with CKD compared to 2.3 in cats with no known conditions (*p* < 0.001). This increase was one of the most prominent indicators of CKD (Table 1). Throughout various elimination events, cats with CKD spent more time eliminating (urination, defecation, or combo). The duration of voiding was also notably longer for these cats during urination events (25.2 s vs. 17.8 s; *p* = 0.015). Interestingly, while the overall event duration for urination was shorter in cats with CKD (58.9 s vs. 70.4 s; *p* = 0.037), this was attributed to significantly reduced post-elimination behaviors, such as covering (14.0 s vs. 22.9 s; *p* = 0.001) and covering intensity or the force that cats are using to move the litter (*p* = 0.036). The cats in the Renal group exhibited a tendency to cover their waste with less intensity. Moreover, cats in the Renal group spent less time covering their waste after urination. The overall time spent covering, regardless of the event, was reduced compared to the Non-Renal group, indicating a broader behavioral shift among the cats in the Renal group. Finally, the cats in the Renal group recorded an increased total weight of output during urination over a 7-day period (1007.4 g vs. 469.8 g; *p* = 0.015), suggesting a rise in urine volume. The respective behavior findings among the seven most important features as selected by RFE from the set of statistically significant features across the Non-Renal and Renal groups stratified by IRIS stages are shown in Figure 3 and Figure 4.

These features collectively highlight a unique behavioral profile in cats with CKD characterized by more frequent urination, longer voiding durations, reduced covering behavior, and increased urine output as compared to cats with no known conditions. While some of these features have been anecdotally reported, this study objectively confirmed differences in behavioral profiles. Some of these behaviors (i.e., reduced covering behavior) are consistent across IRIS stages 1–2 and IRIS stages 3–4 while others are more pronounced in IRIS stages 3–4 compared to IRIS stages 1–2 (i.e., longer voiding durations).

In terms of defecation behavior, cats in the Renal group showed a slightly lower frequency of daily defecation events (0.7 vs. 0.8 per week; *p* = 0.010) and shorter event durations (114.4 s vs. 135.2 s; *p* = 0.025). However, they exhibited longer elimination durations during defecation (32.5 s vs. 20.9 s; *p* = 0.003), again suggesting prolonged posturing or straining. Additional features such as mean total daily visits and transition/covering durations across all visits showed significant differences: the Renal group visits were higher, and the transition/covering durations were briefer compared to the Non-Renal group.

As Table 3 indicates, the model achieved 100% precision for the Renal group in the LOOCV set and 66.7% precision for the Renal group in the validation set. Here, precision represents the ratio of accurate Renal predictions (i.e., the predicted CKD cat was in the Renal group) over total Renal predictions.

In the LOOCV set, all cats predicted to have CKD were indeed in the Renal group. In the validation set, two of the cats who were predicted to have CKD were reported by their caregivers as having “no known conditions.” Furthermore, a 100% recall rate and 95.5% recall rate for the Non-Renal group in the LOOCV set and validation set respectively demonstrate that the majority of cats with no known conditions were accurately identified as such. Here, recall represents the ratio of true Non-Renal predictions (i.e., the cat predicted to have no known conditions was in the Non-Renal group) over the total number of cats in the Non-Renal group.

**Figure 5:**
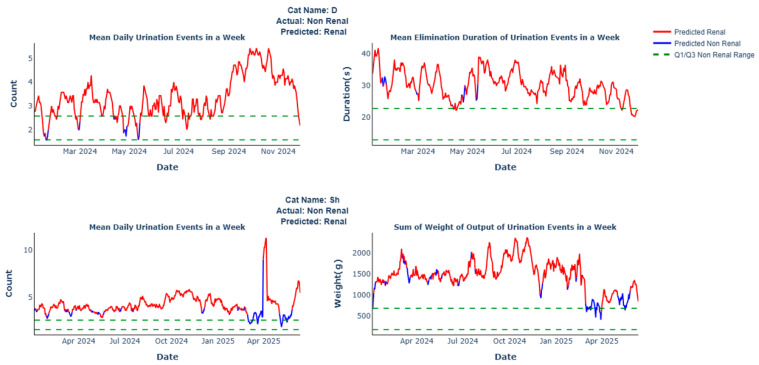
Renal misprediction examples.

The misclassification analyses revealed that six of the cats in the Renal group who did not receive a CKD prediction had a behavioral profile that mirrored cats with no known conditions (IRIS stage 1 (*n* = 1), stage 2 (*n* = 2), stage 3 (*n* = 2), and stage 4 (*n* = 1)). In the validation set, the recall for cats with no known conditions was 95.5%, while the precision of Renal predictions was 66.7%. This lower precision was due to the two cats predicted as having CKD, despite their caregivers self-reporting “no known conditions.” Specifically, Figure 5 shows that cat D exhibited high daily urination and prolonged voiding durations, while cat Sh had a high frequency of daily urinations and an increased weight of output as compared to the Non-Renal group ranges. The behavioral data for these two cats aligns with profiles unique to cats with CKD. It is unknown whether these cats were diagnosed with renal disease after the initial caregiver self-report.

**Figure 6:**
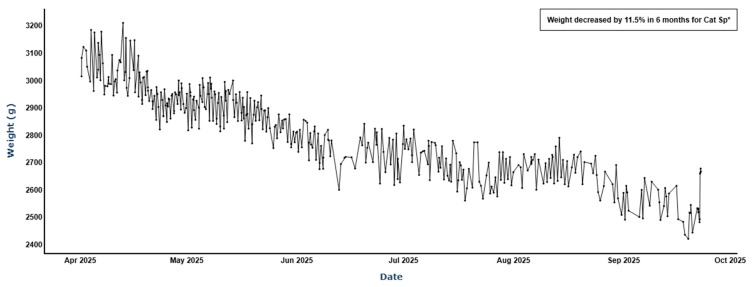
Weight loss trend for cat Sp (11.5% decrease over 6 months), * weight alert triggered.

**Figure 7:**
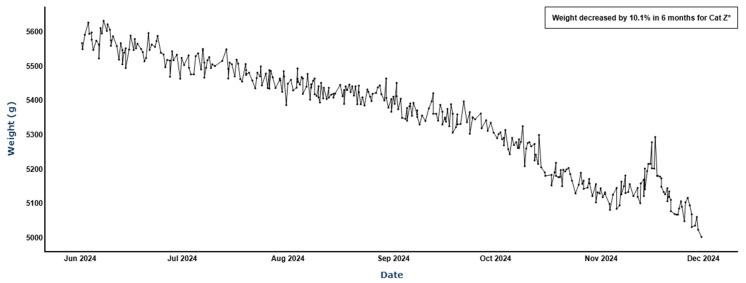
Weight loss trend for cat Z (10.1% decrease over 6 months), * weight alert triggered.

Further, we provide an illustrative example of how this model can be combined with the weight tracking technology of the smart litter box monitor. One of the validation set cats Sp was correctly predicted as having CKD and also showed steady weight decline that was detected by the smart litter box monitor (Figure 6):

Comparatively, cat Z in the validation group was mispredicted as not having CKD despite the caregiver reporting a veterinary CKD diagnosis. Regardless of an absence of the behavioral profile of a CKD cat, this cat had prominent weight loss trends that would have triggered a concerning weight loss alert by the smart litter box monitor. So, while Z may have been “asymptomatic” from the perspective of the machine learning model, a clue into his health state may be more evident by his weight trend as shown in Figure 7:

## 4. Discussion

Due to the model’s optimized precision in predictions for cats with CKD and recall for predictions for cats with no known conditions, the results indicate a reliable, statistically linked behavioral profile for cats with CKD defined as the feature constellation of increased urination frequency, increased urinary output, longer elimination durations, and briefer, less intense post-elimination covering behavior. The model achieved weighted F1-scores of 92.7% (LOOCV) and 89.9% (validation), indicating robust predictive performance and minimization of false positives. The model performance was also consistent across cats representing different IRIS stages, suggesting that the results were not driven by a single disease stage. Key features like increased urination, reduced covering behaviors, and increased urine output were already observable in IRIS stages 1–2 as can be seen in Figure 3 and Figure 4. Overall, the results mirror findings from other studies and general knowledge concerning CKD.

Polyuria (excessive urination) is a common clinical sign preceding CKD diagnosis [14]. In particular, the specific finding of statistically relevant increased weight of urinary output corresponds to the observed symptom of increased urine production which can indicate onset of renal issues [27]. Patterns of higher urination frequency were also found in another study [1]; interestingly, while the most important feature in our dataset was the increased mean daily urination events in a week, the previous study observed that cats with CKD had a higher mean number of urination events per day [1].

Our results also show that while the cats in the Renal group spent more time voiding, the total duration of their elimination events was shorter compared to the cats in the Non-Renal group. This relates to the briefer durations that the cats in the Renal group spent in the post-elimination phase of covering their waste. Further, while it was not statistically significant, the results indicated that the pre-elimination phase was also shorter among the cats in the Renal group. In other words, they also did not spend as much time sniffing or digging prior to eliminating compared to cats in the Non-Renal group. This observation aligns with general understanding of cats living with CKD, especially in later stages, where weight loss and frailty are common symptoms [28,29]. As such, less intense digging/covering behavior among cats in the Renal group is intuitively sound. Musculoskeletal diseases such as osteoarthritis are common comorbidities; however, since the cats in the Non-Renal group were age matched, the differences in covering behavior between the Renal and Non-Renal groups can be attributed to CKD.

Despite the overall success and focus on optimizing precision for CKD predictions to increase the confidence in positive CKD predictions, this design choice did come at the cost of an increased false-negative rate for CKD cats. It was noted that some cats in the Renal group for both the LOOCV and validation sets would not receive a CKD prediction due to a less pronounced behavioral profile. This finding highlights an area for improvement in the predictive model, suggesting that while the accuracy for identified cats with CKD is high, there are some cats with CKD that are not recognized by the model. It is possible that this may indicate they lack overt symptomology or that they are receiving treatment (e.g., medication, supplement, and/or therapeutic diet) whereby expected behaviors are masked. In other cases, treatments such as fluid therapy and prednisone may exacerbate behaviors such as increased urination frequency and output, and treatments for osteoarthritis may improve mobility and alter covering behaviors. Accordingly, model performance should be interpreted within the context of a conservative screening strategy that prioritizes confidence in positive predictions over a comprehensive detector of all renal cases. These findings are expected to generalize primarily to exclusively indoor cats that reliably utilize monitored litter boxes for elimination, where continuous passive data can be consistently captured.

The study was limited by both the small sample size, the limited range of ages of CKD cats, and by the general categorization of “CKD diagnosis” to define the Renal group instead of specific disease stage groupings. A further limitation of this study was that we could not validate the precise health states of the in-home cats beyond confirming a general CKD diagnosis as reported by cat caregivers. However, this limitation does mimic the real-world scenario of the smart litter box monitor technology. Cats listed with “no known condition” may have underlying health issues such as early-stage CKD that are otherwise undetected. Due to the retrospective design constraints, we could not effectively establish a “healthy” control group or validate all health states of the cats with a veterinarian.

General consensus is that CKD is typically only detected very late when the kidney dysfunction is irreversible, and is likely an underdiagnosed health condition [14,30]. Lab diagnostics may not detect azotemia until <75% of renal mass deteriorates [17]. The smart litter box monitor technology, and specifically the machine learning model developed in this study may be a tool for cat caregivers to identify cats demonstrating behavioral profiles consistent with those of CKD cats. The machine learning model combined with the weight tracking component of the smart litter box monitor [18] offers a means for an at-home tool providing continuous data that cat caregivers can bring to their veterinarians to supplement the periodic opportunities to screen for renal health.

While the results of the machine learning model are promising, the limited sample size impacts population-level generalizability. Future work will investigate larger, more balanced cohorts with expanded age ranges for CKD cats across IRIS stages to determine the feasibility of detecting the unique stages and defining the profiles unique to each stage. Further, assessing longitudinal CKD data will help to better track, and potentially predict, the progression of CKD via elimination-related behavioral changes detected by the smart litter box monitor. Larger datasets would also enable us to split the stages and do more careful evaluations within and across CKD groups. Also, while weight trends are incredibly relevant as one of several variables for detecting CKD onset and progression, the machine learning model uses features based on the last 7 days to make a prediction for a single day. For this reason, weight was not a salient feature for the model, but as its own longer-term metric combined with the more precise flagging mechanism of the model, general weight trends from the smart litter box monitor can become more meaningful in the context of CKD detection. Perhaps a future model iteration can combine a behavioral feature set with weight trends. Future work should also investigate cats with other health conditions that may exhibit similar litter box behavior changes to CKD, such as diabetes mellitus, hyperthyroidism, mobility, or musculoskeletal diseases. Successful identification of other disease signatures using similar model development could also further validate this model.

Taken together, the technology of the smart litter box monitor holds promise for effective flagging of behaviors associated with health conditions like CKD while also offering a source for post-diagnostic monitoring via continuous in-home data that leverages a baseline reference to alert caregivers of significant changes to a cat’s typical behaviors. Caregivers of cats with CKD have reported being negatively impacted by necessary changes to daily routines or restrictions on their lives after receiving a diagnosis [31,32]. At-home, non-invasive, passive continuous data collection may also provide an ease of burden or peace of mind around disease management for caregivers.

## 5. Conclusions

CKD is a leading cause of mortality among aging cats rife with challenges associated with early detection and diagnosis due to non-specific symptoms [6,8,9,10]. Despite numerous methodologies employed for timely detection and management of the disease, there remains a critical need for improved detection techniques. This study’s findings highlight the potential of innovative technologies, such as the smart litter box monitor’s machine learning model, to facilitate the identification of behavioral changes indicative of chronic kidney disease. This device, combined with machine learning algorithms, demonstrates promise in providing passively collected longitudinal data to support caregiver and veterinarian decision-making alongside standard veterinary clinical diagnostics.

Moreover, while the model achieved high precision and recall rates, it also revealed limitations, particularly in identifying all CKD cases. This emphasizes the importance of continuous refinement of detection methodologies, including further exploration of specific IRIS stages and the integration of additional health metrics. Future research should focus on expanding datasets and enhancing the model’s capabilities, ensuring that it can effectively support cat caregivers and veterinarians in the detection and management of CKD, thereby contributing to better health outcomes for feline patients.

## Figures and Tables

**Table 1:** Selected features.

Feature Name	Feature Importance
Mean Daily Urination Events in a Week	23.01
Mean Covering Up Intensity of Urination Events in a Week	16.66
Mean Elimination Duration of Urination Events in a Week	15.14
Mean Transition/Covering Duration in a Week	12.56
Mean Transition/Covering Duration of Urination Events in a Week	11.25
Mean Elimination Duration in a Week (Scaled)	10.99
Sum of Weight of Output of Urination Events in a Week	10.40

**Table 2:** Results.

					Model v1	Model v2		Model v3	
Feature Name	Condition	Count	Mean	Std.	Condition *p* Value	Source *p* Value	Condition *p* Value	Source *p* Value	Interaction *p* Value
Mean Daily Non-Elimination Events in a Week	Non-Renal	816	1.2	1.8	0.552	<0.001	**0.008**	<0.001	**0.006**
	Renal	117	1.2	2.1					
Mean Daily Urination Events in a Week	Non-Renal	816	2.3	1.0	**<0.001**	0.641	**<0.001**	0.746	**0.025**
	Renal	117	4.6	2.1					
Mean Daily Defecation Events in a Week	Non-Renal	816	0.8	0.5	0.968	0.146	**0.010**	0.009	**0.002**
	Renal	117	0.7	0.6					
Mean Daily Visits in a Week	Non-Renal	816	4.1	2.5	**<0.001**	0.001	**<0.001**	0.038	**<0.001**
	Renal	117	6.3	4.0					
Mean Event Duration of Non-Elimination Events in a Week	Non-Renal	629	38.2	32.1	0.260	0.002	-	-	-
	Renal	71	54.3	43.5					
Mean Event Duration of Urination Events in a Week	Non-Renal	808	70.4	20.5	**0.037**	0.297	0.305	0.304	0.883
	Renal	117	58.9	18.1					
Mean Entry and Digging Duration of Urination Event in a Week	Non-Renal	726	18.7	8.9	0.321	<0.001	0.361	<0.001	0.716
	Renal	117	13.6	6.1					
Mean Elimination Duration of Urination Events in a Week	Non-Renal	709	17.8	8.3	**0.015**	0.128	0.250	0.179	0.812
	Renal	117	25.2	9.1					
Mean Transition/Covering Duration of Urination Events in a Week	Non-Renal	726	22.9	11.3	**0.001**	0.962	**0.015**	0.810	0.470
	Renal	117	14.0	6.7					
Mean Digging Up Intensity of Urination Events in a Week	Non-Renal	646	0.1	0.2	0.396	0.593	0.964	0.466	0.539
	Renal	108	0.1	0.1					
Mean Covering Up Intensity of Urination Events in a Week	Non-Renal	651	0.2	0.2	**0.036**	0.931	0.400	0.823	0.709
	Renal	112	0.1	0.1					
Mean Event Duration in a Week	Non-Renal	816	78.5	25.4	**0.009**	<0.001	0.122	<0.001	0.966
	Renal	117	64.0	21.1					
Mean Entry and Digging Duration in a Week	Non-Renal	730	19.8	8.2	0.068	0.013	0.133	0.011	0.577
	Renal	117	14.3	5.9					
Mean Elimination Duration in a Week	Non-Renal	713	17.4	7.9	**0.030**	0.026	0.877	0.109	0.192
	Renal	117	24.9	8.2					
Mean Transition/Covering Duration in a Week	Non-Renal	730	24.0	11.7	**<0.001**	0.190	**0.009**	0.277	0.597
	Renal	117	14.7	6.9					
Sum of Weight of Output of Urination Events in a Week	Non-Renal	808	469.8	476.8	**0.015**	0.603	**0.028**	0.368	0.302
	Renal	117	1007.4	634.5					
Mean of Weight of Output of Urination Events in a Week	Non-Renal	808	36.0	29.2	0.315	0.142	0.715	0.074	0.266
	Renal	117	37.9	22.6					
Standard Deviation of Weight in a Week	Non-Renal	598	40.1	47.5	0.374	0.196	0.848	0.178	0.704
	Renal	113	61.8	56.3					
Mean Event Duration of Defecation Events in a Week	Non-Renal	724	135.2	38.7	**0.025**	0.253	**0.032**	0.132	0.277
	Renal	78	114.4	34.1					
Mean Entry and Digging Duration of Defecation Events in a Week	Non-Renal	477	33.9	20.4	0.872	0.961	0.344	0.545	0.323
	Renal	78	28.7	16.4					
Mean Elimination Duration of Defecation Events in a Week	Non-Renal	455	20.9	11.7	**0.003**	0.483	0.061	0.584	0.471
	Renal	78	32.5	12.9					
Mean Transition/Covering Duration of Defecation Events in a Week	Non-Renal	477	39.2	22.3	0.187	0.259	0.629	0.519	0.996
	Renal	78	33.0	31.4					
Sum of Weight of Output of Defecation Events in a Week	Non-Renal	724	129.4	156.9	0.312	0.901	0.232	0.470	0.034
	Renal	97	89.7	140.5					
Mean of Weight of Output of Defecation Events in a Week	Non-Renal	721	31.0	33.5	0.172	0.992	0.842	0.791	0.509
	Renal	95	21.8	25.5					

Bolded numbers correspond to statistically significant values related to condition.

**Table 3:** Model results.

		Total	Predicted Non-Renal	Predicted Renal	Precision	Recall	F1-Score
LOOCV	Non-Renal	72	72	0	92.3%	100.0%	96.0%
	Renal	13	6	7	100.0%	53.8%	70.0%
	Weighted				93.5%	92.9%	92.0%
Validation	Non-Renal	44	42	2	93.3%	95.5%	94.4%
	Renal	7	3	4	66.7%	57.1%	61.5%
	Weighted				89.7%	90.2%	89.9%

## Data Availability

The datasets presented in this article are not readily available due to the proprietary nature of the research and technology development. Requests to access the datasets should be directed to natalie.langenfeld-mccoy@rd.nestle.com.

## Supplementary Materials

The following supporting information can be downloaded at: https://www.mdpi.com/article/10.3390/ani16091319/s1, Table S1: signalment data by group for training cats. Table S2: medication list for 13 training cats.

## References

[1] George Z.M., Quimby J.M., Jones S., Brusach K.B., Rudinsky A.J. (2025). Quantification of defecation frequency in cats with and without chronic kidney disease. J. Feline Med. Surg..

[2] McLeland S., Quimby J., Lappin M.R. (2019). Alpha-enolase staining patterns in the renal tissues of cats with and without chronic kidney disease. Veter. Immunol. Immunopathol..

[3] Sparkes A.H., Caney S., Chalhoub S., Elliott J., Finch N., Gajankayake I., Langston C., Lefebvre H.P., White J., Quimby J. (2016). ISFM consensus guidelines on the diagnosis and management of feline chronic kidney disease. J. Feline Med. Surg..

[4] Chakrabarti S., Syme H.M., Elliott J. (2012). Clinicopathological variables predicting progression of azotemia in cats with chronic kidney disease. J. Veter. Intern. Med..

[5] Kovarikova S. (2015). Urinary biomarkers of renal function in dogs and cats: A review. Veter. Med..

[6] Kongtasai T., Paepe D., Meyer E., Mortier F., Marynissen S., Stammeleer L., Defauw P., Daminet S. (2022). Renal biomarkers in cats: A review of the current status in chronic kidney disease. J. Veter. Intern. Med..

[7] Finch N.C., Syme H.M., Elliott J. (2016). Risk factors for development of chronic kidney disease in cats. J. Veter. Intern. Med..

[8] Vanden Broecke E., Van Mulders L., De Paepe E., Paepe D., Daminet S., Vanhaecke L. (2025). Early detection of feline chronic kidney disease via 3-hydroxykynurenine and machine learning. Sci. Rep..

[9] Marino C.L., Lascelles B.D.X., Vaden S.L., Gruen M.E., Marks S.L. (2014). Prevalence and classification of chronic kidney disease in cats randomly selected from four age groups and in cats recruited for degenerative joint disease studies. J. Feline Med. Surg..

[10] Nassar G., Mahmoud A.R., Mohamed S., El-Sheikh A.K., Bayoumi Y. (2025). Chronic kidney disease in cats. Egypt. J. Veter. Sci..

[11] Cobrin A.R., Blois S.L., Kruth S.A., Abrams-Ogg A.C.G., Dewey C. (2013). Biomarkers in the assessment of acute and chronic kidney diseases in the dog and cat. J. Small Anim. Pract..

[12] Li Q., Cominetti O., Holzwarth J.A., Summers S., Wang X., Dayon L. (2025). Integrated multi-omics analysis of renal metabolism in domestic cats with spontaneous chronic kidney disease. Commun. Biol..

[13] Paepe D., Daminet S. (2013). Feline CKD: Diagnosis, staging and screening—What is recommended?. J. Feline Med. Surg..

[14] Conroy M., Brodbelt D.C., Neill D., Chang Y.-M., Elliott J. (2019). Chronic kidney disease in cats attending primary care practice in the UK: A VetCompassTM study. Veter. Rec..

[15] Bradley R., Tagkopoulos I., Kim M., Kokkinos Y., Panagiotakos T., Kennedy J., De Meyer G., Watson P., Elliott J. (2019). Predicting early risk of chronic kidney disease in cats using routine clinical laboratory tests and machine learning. J. Veer. Intern. Med..

[16] Nickel M.R., Sweet H.M., Lee A., Bohaychuk-Preuss K., Varnhagen C., Olson M. (2022). A saliva urea test strip for use in feline and canine patients: A pilot study. J. Veter. Diagn. Investig..

[17] Jepson R.E., Brodbelt D., Vallance C., Syme H.M., Elliott J. (2009). Evaluation of predictors of the development of azotemia in cats. J. Veter. Intern. Med..

[18] Snow L., Langenfeld-McCoy N., Dussan H., Arndt O., Schoeneck N., Thomas S., McGowan R.T.S. (2025). Enhancing cat care: Unveiling the technology of intelligent litter box monitoring. Appl. Anim. Behav. Sci..

[19] R Core Team (2023). R: A Language and Environment for Statistical Computing.

[20] Brooks M.E., Kristensen K., van Benthem K.J., Magnusson A., Berg C.W., Nielsen A., Højsgaard S. (2017). glmmTMB balances speed and flexibility among packages for zero-inflated generalized linear mixed modeling. R J..

[21] Ellis J.J., McGowan R.T.S., Martin F. (2017). Does previous use affect litter box appeal in multi-cat households?. Behav. Process..

[22] McGowan R.T.S., Ellis J.J., Bensky M.K., Martin F. (2017). The ins and outs of the litter box: A detailed ethogram of cat elimination behavior in two contrasting environments. Appl. Anim. Behav. Sci..

[23] Python Software Foundation (2023). Python 3.12. https://www.python.org/downloads/release/python-3120/.

[24] Oliphant T.E. (2015). A Guide to NumPy.

[25] Pedregosa F., Varoquaux G., Gramfort A., Michel V., Thirion B., Grisel O., Duchesnay É. (2011). Scikit-learn: Machine learning in Python. J. Mach. Learn. Res..

[26] Dorogush A.V., Ershov V., Gulin A. (2018). CatBoost: Gradient boosting with categorical features support. arXiv.

[27] Grauer G.F. (2005). Early Detection of renal damage and disease in dogs and cats. Veter. Clin. N. Am. Small Anim. Pract..

[28] Greene J.P., Lefebvre S.L., Wang M., Yang M., Lund E.M., Polzin D.J. (2014). Risk factors associated with the development of chronic kidney disease in cats evaluated at primary care veterinary hospitals. J. Am. Veter. Med. Assoc..

[29] Pye C.R., Dowgray N.J., Eyre K., Pinchbeck G., Biourge V., Moniot D., Comerford E., German A.J. (2025). Longitudinal changes in bodyweight, body condition, and muscle condition in ageing pet cats: Findings from the Cat Prospective Ageing and Welfare Study. Front. Veter. Sci.

[30] Chen H., Dunaevich A., Apfelbaum N., Kuzi S., Mazaki-Tovi M., Aroch I., Segev G. (2020). Acute on chronic kidney disease in cats: Etiology, clinical and clinicopathologic findings, prognostic markers, and outcome. J. Veter. Intern. Med..

[31] Elliott J., Reyes-Hughes H., Hibbert A., Blackwell E., Finch N.C. (2025). Owners’ experiences of caring for cats with chronic kidney disease in the UK. J. Feline Med. Surg..

[32] Boyd L.M., Langston C., Thompson K., Zivin K., Imanishi M. (2008). Survival in Cats with Naturally Occurring Chronic Kidney Disease (2000–2002). J. Vet. Intern. Med..

