# Supplementary material for: Enhancing Detection of Feline Chronic Kidney Disease Through Smart Litter Box Monitoring
Source: Animals (Basel). 2026 Apr 25;16(9):1319. doi: 10.3390/ani16091319 (PMC13162653; doi:10.3390/ani16091319)
Supplement: Supplementary file 1 [file animals-16-01319-s001.zip › animals-4181628-supplementary.pdf]

Supplementary Materials:

Table S1: signalment data by group for training cats

	Renal (n = 13)	Non-Renal (n = 72)
**Age (years)**		
Mean ± SD	12.43 ± 4.19	7.47 ± 4.20
Median (IQR)	11.80 (11.40 - 13.40)	7.65 (3.92 - 10.30)
Range	4.20 - 20.00	0.80 - 23.00
**Sex, n (%)**		
Male	5 (38.5%)	41 (56.9%)
Female	8 (61.5%)	31 (43.1%)

Table S2: medication list for 13 training cats

Cat	Stage	Medications
1	Stage 4	Solensia; Green study capsule for Porus One
2	Stage 1	none
3	Stage 2	Miralax 1/4 tsp once EOD, Gabapentin 100 mg for appointments, Green/Orange Capsule (PORUS One/Placebo)
4	Stage 3	Mirataz 1.5 in transdermal every 2 days, SQ fluids 50 ml twice a week, amlodipine 0.625 mg PO SID, gabapentin if staying at sister's house, Solensia once a month with rDVM, potassium gluconate 1/2 tsp SID, aluminum hydroxide powder 1 scoop BID, Green study capsule for Porus One
5	Stage 4	SQ fluids 200 ml twice week, Orange study capsule SID (PORUS One/Placebo)
6	Stage 2	none
7	Stage 2	Mirataz transdermal EOD, Gabapentin 10 mg before appt, Porus One Orange Capsule SID (PORUS One/Placebo)
8	Stage 3	Aluminum Hydroxide 64 mg/mL Oral Suspension: 2.4mL (160mg total daily, split between all meals), Mirtazapine transdermal ointment: applied to ear 2-3 times per week, SQ fluids: 100mL twice a week, Cerenia 16mg: 1/4 tab (1mg/kg) PO PRN - stopped 5/29/23, Darbepoetin 25mcg/ml: 0.15mL (0.9mcg/kg) SQ ~2 to 4 weeks; Gabapentin for appointments
9	Stage 3	prednisolone 5 mg PO SID for allergies, mirataz 1/4-1/2 inch transdermal every 3 days, gabapentin ~25 mg PO for appointments
10	Stage 3	Potassium gluconate (RenaPlus) 8 mEq/tsp 1/4 tsp PO q24h, Aluminum Hydroxide 2250 mg/tsp: 1/16 tsp PO q12h, Amlodipine 1.25 mg PO q24 hours, Darbepoietin q4-6 weeks, HydraCare as a treat, PorusOne/Placebo for study, Gabapentin 50 mg/ml: 1 ml PO 1-2 hours prior to vet visits
11	Stage 1	Methimazole 5mg Daily Long term
12	Stage 2	Gabapentin (50 mg) daily Long term
13	Stage 3	Mirataz Transdermal: 1.5 inch applied to pinna PRN, Porus One: 1 packet mixed on food once daily
